# The impact of the introduction of a multi-professional altered airway ward round on improving patient safety

**DOI:** 10.1186/2197-425X-3-S1-A279

**Published:** 2015-10-01

**Authors:** A Young, I Naldrett, L Carey, V Lutchmee, C Soares, T Pemberton, N Maistry, H Raubenheimer, A Gunawardena Daamayanthi, A Pendilhe, L Allibone, M Dusmet, A Ghori, S Jordan, M Griffiths, M White

**Affiliations:** Royal Brompton and Harefield NHS Foundation Trust, Speech and Language Department, London, United Kingdom; Royal Brompton and Harefield NHS Foundation Trust, Adult Intensive Care Medicine, London, United Kingdom; Royal Brompton and Harefield NHS Foundation Trust, Physiotherapy, London, United Kingdom; Royal Brompton and Harefield NHS Foundation Trust, Respiratory Medicine, London, United Kingdom; Royal Brompton and Harefield NHS Foundation Trust, High Dependancy Unit, London, United Kingdom; Royal Brompton and Harefield NHS Foundation Trust, Thoracic Surgery, London, United Kingdom; Royal Brompton and Harefield NHS Foundation Trust, Nursing Development, London, United Kingdom; Royal Brompton and Harefield NHS Foundation Trust, Anaesthesia, London, United Kingdom

## Introduction

Multidisciplinary tracheostomy teams have been implemented in acute hospital settings for over ten years. There is evidence that this initiative contributes to a reduction in total tracheostomy time and an increase in speaking valve use for patients leading to improved quality of life [1]. The recent *NCEPOD safe trache* report recommended a number of safety factors to maintain patient wellbeing in this cohort [2]. We investigated whether the introduction of a multidisciplinary tracheostomy team had an impact on the reduction of adverse incidents.

## Objectives

To determine the tracheostomy rounds impact the incidence of adverse incidents in tracheostomised patients.

## Methods

Data was prospectively collected over a 3-month period on 64 patients on the ICU, HDU, and general ward (January 2015 to March 2015). Data collected included the access to availability of a difficult airway trolley and fiberoptic bronchoscopy, presence of emergency equipment at the bedside, identifiable tracheostomy information, the presence of emergency airway management in the event of tracheostomy emergency, the presence of a weaning and nutrition plan.

## Results

A total of 64 patient contact episodes were recorded. 16 incidences of latent patient safety issues were identified (16/64; 25%). January (n = 8; 12.5%), February (n = 6; 9.4%) and March (n = 2; 3.1%). Emergency airway plan related (n = 11; 17.1%), missing equipment (n = 8; 12.5%). The number incidence of blocked or dislodged tracheostomy in this period was 0. All patients reviewed had an appropriate weaning and nutrition plan.

## Conclusions

The introduction of a multi-disciplinary tracheostomy outreach team has led to a reduction in the latent critical incidents associated with tracheostomised patients. This audit highlights that the presence of a MDT tracheostomy ward round has a positive impact on patient safety.
